# Armored RNA technology as a clinical diagnostics tool for future pandemic preparedness

**DOI:** 10.71150/jm.2510016

**Published:** 2026-02-28

**Authors:** Jin Hao Tan, Prashant Mainali, Wei Zhang, Dave Siak-Wei Ow

**Affiliations:** 1 Microbial Cell Bioprocessing, Bioprocessing Technology Institute (BTI), Agency for Science, Technology and Research (A*STAR), Singapore 138668, Republic of Singapore; 2 Downstream Processing, Bioprocessing Technology Institute (BTI), Agency for Science, Technology and Research (A*STAR), Singapore 138668, Republic of Singapore

**Keywords:** armored RNA, *E. coli* expression, pandemic preparedness, RT-PCR, MS2 bacteriophage

## Abstract

The COVID-19 pandemic highlighted the critical role of reliable molecular diagnostics in outbreak response and the vulnerabilities of existing systems to delays and reagent instability. Armored RNA technology, which packages RNA within bacteriophage-derived capsids, offers a robust solution by combining nuclease resistance, safety, and versatility into a single platform. Armored RNA has become a trusted internal and external control for RT-qPCR and RT-LAMP, enabling accurate detection across a wide range of viral pathogens. Also, recent advances in alternative expression systems, such as plant-based and cell-free platforms, as well as the use of more stable scaffolds from bacteriophage Qβ, are enhancing yield, stability, and accessibility of armored RNA. Engineering innovations, including capsid polymorphism and optimized downstream purification, further improve efficiency and broaden possible applications. Looking ahead, armored RNA holds promise not only as a diagnostic standard but also as a delivery vehicle for vaccines and therapeutics. Encapsulation of self-amplifying RNA, small interfering RNA, or microRNA could open new pathways for rapid-response vaccines and targeted therapies, aligning this technology with the future of precision medicine. By uniting stability, scalability, and adaptability, armored RNA represents a critical component of global health preparedness, with the potential to strengthen diagnostic resilience and accelerate biomedical countermeasures in future pandemics.

## Introduction

COVID-19 was declared a global pandemic on March 11, 2020 by the World Health Organization (WHO) (WHO, 2020). According to WHO, a pandemic is defined as “the worldwide spread of a new disease.” With this recent pandemic still fresh in the public’s minds, it is important to renew efforts on plans to prevent future disease outbreaks from reaching the pandemic status. In pandemic preparedness, there are several elements such as – surveillance & early detection (Inbanathan et al., 2024; Lee et al., 2023), laboratory & diagnostic capacity (Inbanathan et al., 2024; Pabbaraju et al., 2020), medical countermeasures (Jester et al., 2018; Johnson et al., 2022), supply chains & logistics (Kumar et al., 2024; Rozhkov et al., 2022; Wright et al., 2024; Xie et al., 2025). Among these, the establishment of improved public health surveillance will allow for better control of disease spread.

The field of clinical diagnostics has become increasingly reliant on sensitive molecular assays for the detection of viral ribonucleic acid (RNA), such as reverse transcription PCR (RT-PCR). RT-PCR assays are central to managing pandemics because they provide rapid, sensitive, and specific detection of viral RNA, enabling early diagnosis, guiding patient isolation and treatment, and supporting large-scale epidemiological surveillance. Their accuracy and reliability, however, depend heavily on the use of stable, well-characterized viral RNA controls and standards. A key challenge lies in the intrinsic fragility of naked RNA, which is highly susceptible to degradation by ribonucleases (RNases), threatening the integrity of RT-PCR controls throughout synthesis, storage, and transport. This limitation is magnified during pandemics, where massive testing efforts require controls that remain stable across long supply chains and variable storage conditions. Addressing these challenges is essential to ensure the reproducibility and robustness of RT-PCR testing, which underpins both clinical decision-making and public health interventions

To address this critical gap, Armored RNA (aRNA) technology was developed in 1998 by Pasloske and his colleagues (Pasloske et al., 1998). This innovation provides a method to protect RNA molecules from enzymatic degradation, ensuring their stability and integrity for use as reliable controls in molecular assays (Pasloske et al., 1998).

**Fig. 1.**
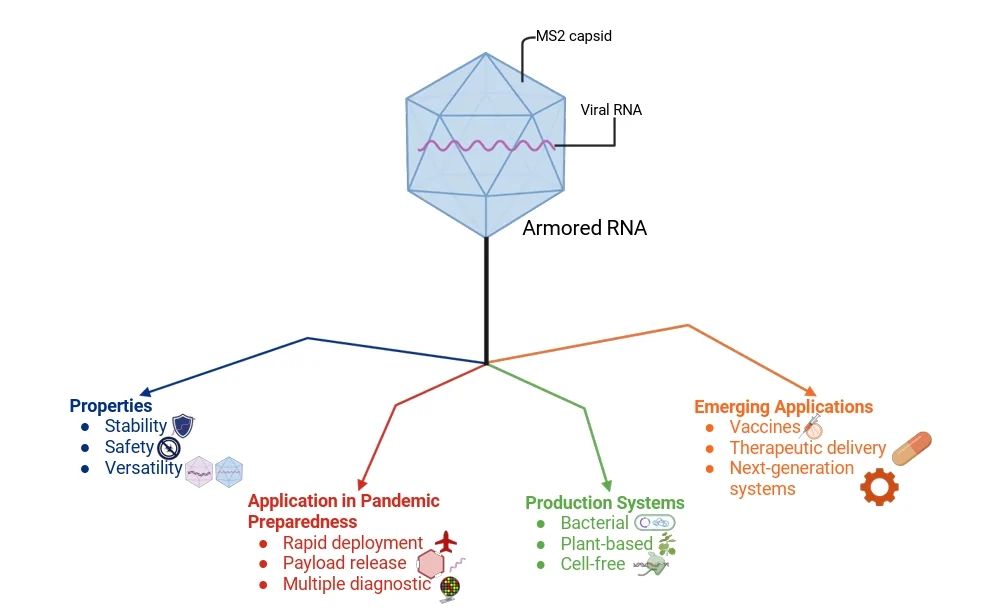
Schematic Overview of Armored RNA and its Key Attributes. Schematic illustration of an armored RNA particle composed of viral RNA encapsidated within an MS2 bacteriophage icosahedral capsid. Arrows highlight the main themes discussed in the review: (i) *Properties* – notably stability, biosafety, and versatility in packing diverse nucleic acid types; (ii) *Applications in pandemic preparedness* – such as rapid deployment and payload release of molecular standards, and multiplex RT-PCR diagnostics; (iii) *Production systems* – including bacterial expression, plant-based expression, and cell-free assembly; and (iv) *Emerging applications* – including roles of aRNA in vaccines, therapeutic RNA delivery, and next-generation engineered capsid systems. Created with BioRender.com.

In this review, we describe aRNA technology and discuss its role in pandemic preparedness, spanning established and emerging production platforms as well as applications beyond diagnostics (Fig. 1). Although previous reviews have extensively addressed the foundational chemistry of MS2-based armored RNA controls, the field has undergone significant transformation following the COVID-19 pandemic. Accordingly, this review focuses on the transition from traditional *E. coli*-based systems to newer production strategies, including plant-based and cell-free approaches, and highlights the expanding potential of aRNA as a platform for therapeutic delivery and self-amplifying RNA vaccine development.

## The Armored RNA (aRNA) Technology

### Structure and assembly in a canonical MS2-based system

Armored RNA technology leverages the self-assembling properties of bacteriophage coat proteins to create a protective shell around a specific RNA molecule. The most established aRNA system uses the coat protein from the MS2 bacteriophage, a single-stranded RNA virus that infects *Escherichia coli* (Liang et al., 2025; Mikel et al., 2015). The production process typically involves an *E. coli* expression system containing an inducible plasmid (Pasloske et al., 1998). This plasmid is engineered to express two key components: the MS2 coat protein and the target RNA sequence of interest. Crucially, the target RNA includes a specific stem-loop structure known as the pac site, which is the MS2 packaging signal. The high-affinity interaction between the MS2 coat protein dimers and the pac site RNA stem-loop initiates a cooperative self-assembly process (Peabody, 1993), resulting in the formation of a non-infectious, icosahedral virus-like particle (VLP) with the target RNA encapsulated inside (Pasloske et al., 1998; Peabody et al., 2021; Wei et al., 2008a). This self-assembly completely protects the internal RNA from RNase activity. It has been reported that a single liter of *E. coli* culture can yield up to 10^15^ aRNA particles, highlighting the immense scalability of this production system (Pasloske et al., 1998).

### Key properties and advantages of aRNA

**Exceptional nuclease resistance and stability:** The primary advantage of aRNA is its remarkable stability. The protein capsid forms a physical barrier that renders the encapsulated RNA resistant to degradation by nucleases (Gholami et al., 2018; Pasloske et al., 1998). Studies have shown that while naked RNA degrades rapidly, aRNA particles remain stable when incubated in human serum or plasma (Gholami et al., 2018; Pasloske et al., 1998; Wei et al., 2008b). They have demonstrated no loss of signal using a human immunodeficiency virus (HIV-1) RT-PCR assay after incubation in plasma and have been shown to withstand at least five freeze-thaw cycles (Pasloske et al., 1998). Further studies have confirmed long-term stability, with aRNA particles remaining stable for over two months in newborn calf serum at temperatures of 4, 25, and 37°C (Wei et al., 2008b). Plant-produced aRNA mimics have shown similar robustness, remaining stable after five freeze-thaw cycles or after 16 h at 55°C (Peyret et al., 2022). This enhanced stability allows for longer shelf-life of RNA controls and alleviates the need for a stringent cold chain, which is a critical logistical advantage for global distribution during a pandemic.

**Enhanced safety of armored RNA:** Unlike live attenuated or inactivated viral controls, aRNA particles are entirely non-infectious because they lack the genetic material required for viral replication. This eliminates the biosafety risks associated with handling live pathogens, making them safer to produce and handle in a wide range of laboratory settings, including those with lower biosafety levels (BSL-1 or BSL-2). Over the past two decades, numerous studies have reinforced the safety profile of aRNA. Early work demonstrated that encapsidated RNA particles are nuclease resistant, stable under routine storage and transport conditions, and non-infectious, making them practical alternatives to hazardous viral controls (Hietala and Crossley, 2006; Yu et al., 2008). Subsequent developments expanded this approach to plant virus–based encapsidation systems, such as cowpea mosaic virus (CPMV), which produced highly stable RNA particles that were noninfectious to humans and animals and could be safely used as external controls in diagnostic assays (Madi et al., 2015).

The COVID-19 pandemic further highlighted the utility of aRNA as a safe surrogate for high-risk pathogens. Studies reported the successful packaging of SARS-CoV-2 target sequences into both plant virus–like particles and bacteriophage MS2 capsids, confirming that these constructs could be deployed as standardized positive controls for RT-qPCR without introducing any infectious risks (Goncharova et al., 2021; Peyret et al., 2022). Importantly, these systems combined biosafety, stability, and scalability, enabling global distribution of controls without requiring high-containment facilities. Taken together, the literature establishes armored RNA as a biosafe, stable, and logistically practical diagnostic surrogate. By providing noninfectious controls that withstand nuclease degradation and routine handling, aRNA reduces both biosafety concerns in the laboratory and biosecurity risks during distribution, supporting its adoption across diverse diagnostic platforms (Goncharova et al., 2021; Hietala and Crossley, 2006; Madi et al., 2015; Peyret et al., 2022; Yu et al., 2008).

**Versatility of armored RNA technology for all types of viruses:** Furthermore, the technology is highly modular. The production system can be easily adapted to encapsulate virtually any RNA or deoxyribonucleic acid (DNA) sequence of interest, provided it is within the size constraints of the capsid and contains the necessary packaging signal. This versatility has been demonstrated by the development of aRNA controls for a wide array of viral targets (Table 1) since its initial proof-of-concept with HIV-1, including hepatitis C virus (HCV), enterovirus, influenza viruses, and coronaviruses. Besides single-stranded RNA, the technology has been further expanded to produce armored DNA, encapsulating double-stranded DNA sequences from hepatitis B virus (HBV) (Crone and Freemont, 2022) and human papillomavirus (HPV) (Zhang et al., 2015a) and single-stranded DNA (Hashemi et al., 2021; Zilberzwige-Tal et al., 2021).

### Use of aRNA in virus diagnostic

In routine virus diagnostics, aRNA are used widely as external positive controls, quantitative standards, and internal process controls. As external controls or standards, they provide well-defined copy numbers for generating standard curves and assessing assay linearity and limit of detection (LOD) for RT-PCR or other nucleic acid amplification tests (NAATs) (Yu et al., 2008; Zhao et al., 2007). As aRNA (unlike bare RNA) survive extraction and RT steps, they allow the lab to measure the whole workflow (extraction → RT → amplification) rather than only the amplification step. As internal process controls, aRNA is spiked into patient samples to reveal extraction failures or RT-PCR inhibition in individual specimens, which reduces false negatives (Stevenson et al., 2008). These practical roles have been demonstrated for multiple viral targets (HIV, HCV, influenza, SARS-CoV, measles and others) and for different assay formats, including multiplex real-time RT-PCR (Yu et al., 2008).

aRNA is also used for proficiency testing, assay development, and supply-chain use. Due to aRNA being non-infectious surrogates that behave like real viruses in extraction and detection steps, aRNA panels are frequently used in external quality assessment (EQA) and inter-laboratory comparisons to evaluate laboratory performance without sending infectious material (Zhang et al., 2015b). They are also convenient for assay validation and method transfers and for rapid kit development during outbreaks because they can be produced to contain any target sequence of interest (Crone et al., 2020). Finally, their relative biosafety and stability ease storage and shipping, lowering logistical burdens during large-scale testing programs (Hietala and Crossley, 2006; Pasloske et al., 1998).

## Application of aRNA in Pandemic Preparedness

### Rapid development and deployment of diagnostic controls

The modular nature and established production protocols for aRNA allow for the rapid development of controls for emerging pathogens. The production platform, typically relying on standardized plasmids and *E. coli* expression systems, is a key reason for this speed. Once a novel pathogen is identified and its genome sequenced, a synthetic gene fragment corresponding to a key diagnostic target can be designed and synthesized within days. This fragment, containing the necessary pac site, is then cloned into an expression plasmid to produce the aRNA control (Lin et al., 2017).

This capability was demonstrated during the COVID-19 pandemic. Following the publication of the SARS-CoV-2 genome, researchers were able to develop and validate specific aRNA controls within months. These controls, encapsulating RNA sequences for genes like nucleocapsid (N), envelope (E), and RNA-dependent RNA polymerase (RdRp), became indispensable for the global rollout of RT-qPCR diagnostics, ensuring that laboratories could validate their assays and report results with confidence (Goncharova et al., 2021). Besides RT-qPCR, reverse transcription loop-mediated isothermal amplification (RT-LAMP) was a fast and inexpensive method for detecting coronavirus disease 2019 (COVID-19). aRNA or VLPs produced using capsids from bacteriophage Qβ and plant virus cowpea chlorotic mottle virus (CCMV) successfully encapsidated truncated SARS-CoV-2 RNAs and functioned as full-process controls for both RT-LAMP and RT-qPCR assays (Chan et al., 2021). This ability to quickly generate stable, reliable, and safe controls for the ease of new assay development is a cornerstone of an effective response to a new viral threat.

### aRNA payload release for diagnostic deployment

For aRNA to function as a control in an assay like RT-qPCR, the protected RNA must be efficiently released from its protein shell. This critical step is integrated into the standard nucleic acid extraction protocols that precede most molecular assays, requiring no modification to existing workflows (Beld et al., 2004). Standard viral RNA extraction kits utilize strong protein-denaturing agents, such as guanidinium thiocyanate, often in combination with a heat lysis step (Boom et al., 1990). Guanidinium thiocyanate is a powerful chaotropic agent that works by disrupting the hydrogen bonds and hydrophobic interactions that maintain the protein capsid's tertiary structure (Boom et al., 1999). This causes the VLP to disassemble, releasing the internal RNA into the lysis buffer. The same agent also inactivates any RNases present, protecting the now-vulnerable RNA from degradation (Boom et al., 1990). Because the aRNA particle remains intact until this deliberate lysis step, it serves as an ideal full-process control, verifying the efficacy of the entire diagnostic workflow from sample extraction through to amplification and detection (Donia et al., 2005).

### Application in multiplex diagnostics

The versatility of aRNA technology makes it exceptionally well-suited for multiplex diagnostic assays, which simultaneously detect multiple pathogens in a single reaction. This is particularly valuable during pandemics and seasonal outbreaks where different viruses (e.g., influenza, RSV, and coronaviruses) can cause clinically indistinguishable symptoms (Costa et al., 2023). Creating reliable controls for such assays can be complex and costly, often requiring the addition of multiple separate control materials.

Armored RNA provides an elegant solution by allowing for the creation of a single, chimeric RNA molecule that contains the specific target sequences for all pathogens in the multiplex panel. This multi-target RNA can then be encapsulated into a single aRNA particle. For example, a single aRNA construct was developed to serve as a comprehensive positive control for a multiplex RT-PCR assay detecting Influenza A virus, Influenza B virus, and human respiratory syncytial virus (RSV) (Hymas et al., 2010). Another multiplex RT-PCR control for Influenza A, Influenza B, and SARS-CoV with high sensitivity was also developed (Yu et al., 2008). Similarly, another study developed a multiplex aRNA control for three key viruses causing hand, foot, and mouth disease (Song et al., 2011). This single control particle validates the extraction, reverse transcription, and amplification steps for all viral targets simultaneously, simplifying quality control, reducing the potential for pipetting errors, and lowering costs (Song et al., 2011; Yu et al., 2008). Consistent with the robust stability profile described earlier, this multiplex control maintained integrity for at least two weeks at room temperature, confirming its utility for diverse laboratory settings (Yu et al., 2008).

## Alternative Production Systems

**Fig. 2.**
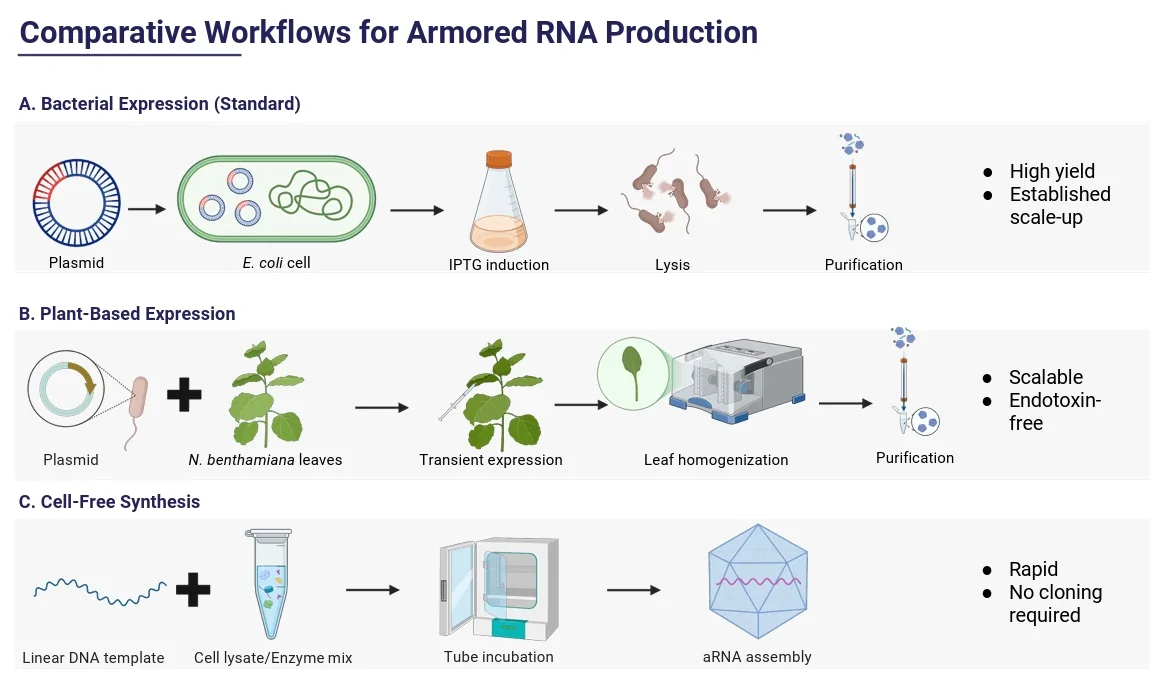
Comparison of Armored RNA Production Platforms. (A) The canonical *E. coli* expression system involves plasmid transformation and induction, offering high yields with established scale-up processes. (B) Plant-based systems utilize *Agrobacterium*-mediated transient expression in *Nicotiana benthamiana*, producing stable particles free of bacterial endotoxins. (C) Cell-free systems allow for rapid assembly from linear DNA templates without the need for cell culture, though at a higher reagent cost. Created with BioRender.com.

Beyond the canonical *E. coli* system, alternative production platforms are being explored to overcome certain limitations and to diversify manufacturing options (Fig. 2).

### Plant-based expression system

A notable example is a plant-based expression system. This method uses the CPMV as the source of coat proteins, which are transiently expressed in the leaves of plants like *Nicotiana benthamiana*. These non-infectious coat proteins then self-assemble around a target RNA, such as a diagnostic region from the SARS-CoV-2 genome, to form stable VLPs (Peyret et al., 2022). This plant-based system offers distinct advantages (Table 2). It provides a highly scalable production platform, with yields reported to be between 0.2 to 1 mg of purified aRNA per gram of fresh-weight leaf tissue. Crucially, plant-derived particles maintain the thermal and nuclease stability characteristic of the bacterial-derived aRNA described earlier (Peyret et al., 2022).

### Cell-free production systems

Besides the above-mentioned production systems, cell-free production for aRNA is also possible as detailed in patents published in 2016 (Zhang et al., 2016b) and 2019 (Zhang et al., 2019). Cell-free aRNA production allows for minimal DNA contamination in the product as there are no cells involved, and the linear DNA input in the system is destroyed by DNase. This method also allows for rapid setup as there is less cloning involved and cell growth is not needed. aRNA produced is similar to that of the *E. coli* system, though the reagents are more costly compared to the other two methods. Despite achieving similar total error rates as *E. coli* systems, well-optimized cell-free systems require reagents of high-quality, as well as additional fidelity-enhancing factors like GreA or GreB which are naturally found in *E. coli*, therefore increasing costs (de Maddalena et al., 2016; Hsu et al., 1995).

## Limitations and Considerations

While highly effective, aRNA technology is not without its limitations. The most significant constraint is the size of the RNA that can be efficiently packaged, which is determined by the internal volume of the VLP. For the standard MS2 bacteriophage system, Pasloske and his colleagues determined that up to 500 bp of RNA could be efficiently packed into aRNA (Pasloske et al., 1998). This makes it unsuitable for encapsulating full-length genomes of larger viruses to act as a complete genomic control. At least 2 or more aRNA have to be designed and prepared for the quality control of such virus detection testing (Sun et al., 2013; Zhang et al., 2015b, 2016a). However, Huang et al. (2006) successfully packaged a 1,200 bp RNA into aRNA particles. Researchers were then able to further pack larger lengths of RNA into the particles, 2,200 bp in 2008 (Wei et al., 2008b), and 4,900 bp in 2017 (Lin et al., 2017).

Beyond packaging size, product purity remains a challenge for the dominant *E. coli* production platform (Liu et al., 2024). Bacterial expression systems naturally contain high levels of endotoxins, which are difficult to remove without complex downstream processing (Huang et al., 2017). While this is less critical for in vitro diagnostics, it presents a significant barrier for the emerging therapeutic and vaccine applications discussed later, necessitating the exploration of endotoxin-free alternatives like plant-based systems (Hemmati et al., 2022).

Cost and scalability also vary significantly across production methods. While cell-free synthesis offers the fastest turnaround time by eliminating cell culture, it relies on expensive reagents and fidelity-enhancing factors (e.g., GreA, GreB) (de Maddalena et al., 2016; Hsu et al., 1995). This makes it less economically viable for large-scale manufacturing compared to the established, high-yield bacterial fermentation processes (Hunt et al., 2025).

Finally, assembly heterogeneity can complicate standardization. Efforts to engineer larger capsids (e.g., *T* = 4 geometry) to accommodate bigger payloads often result in a mixture of capsid sizes dependent on the ratio of asymmetric dimers (Asor et al., 2020; Guo et al., 2024). This lack of uniformity can impact the consistency required for precise quantitative standards.

## Future Directions and Emerging Applications

Research and innovation in aRNA technology are ongoing, with new applications emerging that extend beyond its role as diagnostic control.

### Vaccine development

A significant area of development is the integration of aRNA principles with self-amplifying RNA (saRNA) technology. saRNA is an engineered synthetic nucleic acid capable of replicating within cells without producing viral particles. Enhanced adaptive immune response can be induced due to sustained antigen production and amplification of RNA in the transfected cell from a very small dose (Silva-Pilipich et al., 2024; Vallet and Vignuzzi, 2025). However, saRNA are incredibly unstable as it can be degraded by RNases, in vivo endonucleases and 5’ exonucleases, protein cofactors such as helicases, polymerases, and chaperones, oxidation, and hydrolysis in alkaline environment (Uddin and Roni, 2021). Encapsulating saRNA within a protective VLP not only shields it from degradation but can also enhance its delivery to immune cells and act as a natural adjuvant to boost the immune response, potentially leading to more potent and dose-sparing vaccines as tested in the vaccination of mice (Biddlecome et al., 2019).

### Therapeutic delivery

Modifying VLP coat proteins to enable tissue- or cell-specific targeting, transforms aRNA into a potential delivery vehicle for therapeutic RNAs. By genetically fusing or chemically attaching targeting ligands or epitopes (such as antibodies or peptides) to the capsid surface, these particles can be engineered to bind specifically to receptors on target cells, such as cancer cells. This could enable the targeted delivery of payloads like small interfering RNAs (siRNAs) or antisense oligonucleotides, combining the stability of the armored platform with precision therapeutic action (Chung et al., 2023; Galaway and Stockley, 2013). MicroRNA (miRNA) is a noncoding 20 to 24 nucleotides long RNA strand, able to bind to 3’ untranslated region of a targeted messenger RNA (mRNA), leading to translational suppression by degradation of mRNA. In one study, *E. coli* was used to express both MS2 capsid and miRNA. The capsid containing miRNA is then conjugated with cell-penetrating peptides (CPP) to enable the delivery of miRNA to target cells, allowing for successful gene therapy of human cells, and mice (Pan et al., 2012).

### Next-generation systems

To overcome the payload size limitations of the MS2 capsid systems, strategies being explored include developing methods for polymorphism of VLPs, development of assembly mechanisms, and alternative coat proteins from other viruses. Firstly, generating MS2-like capsids with *T* = 4 geometry instead of the usual wild-type *T* = 3 geometry was proven to be possible. MS2 VLPs with *T* = 4 geometry are comprised of a total of 120 CP dimers instead of 90 CP dimers, having a larger structure and therefore internal volume to load cargo. However, in current methods, the percentage of *T* = 3 and *T* = 4 MS2 VLPs formed are dependent on the fraction of asymmetric dimers of CP (Biela et al., 2022). Next, Wei et al. (2008a) demonstrated that by increasing the number of pac sites from one to two, an 1891-nt exogenous target RNA was packaged successfully, instead of only approximately 1200-nt targets with a single pac site. Finally, alternative coat proteins from other viruses allow for more optimized production of aRNA with characteristics like improved stability or better CP engineering potential as compared to MS2 capsids. CP from other bacteriophages like AP205 and Qβ improve VLP stability due to differences in structure of CP. Looking at the CP, the N-terminal-most β-strand of MS2 is moved to the C-terminus in AP205 (Peabody et al., 2021). This means that the CP dimer has an exposed N- and C-terminus, which gives it the ability to accommodate long insertions at either end, while maintaining capsid structure. Insertions such as spike receptor binding motif of the SARS-CoV-2 to the CP allows the VLPs to be used as a vaccine molecule, inducing high levels antibodies in vaccinated mice (Liu et al., 2021). Qβ CP contains two cysteine residues as compared to MS2 CP. This allows for improved thermal stability by covalently linking individual CP monomers to either hexameric or pentameric subunits while MS2 particles are less covalently cross-linked due to buried cysteine molecules, unable to form these same inter-subunit disulfide bonds (Yao et al., 2019). As a result, aRNA produced with Qβ capsid were significantly more stable at the tested temperatures of -20, 4, and 45°C. An example given was that after 60 days incubation at 4°C, RNA copy numbers of aRNA made of Qβ capsid and MS2 capsid decreased by 12.0% and 38.9% respectively.

### Production efficiency

In a paper documenting stepwise improvements to downstream processing processes, improved aRNA purity, yield, and lot-to-lot consistency was achieved by combining improved precipitation, chromatographic polishing, and buffer optimization. Hashemi et al. (2021) reported that 100 mM NaNO_3_-Tris buffer with pH of 8 is an optimal MS2 VLP production buffer amongst the other buffers tested, to prevent VLPs aggregation, thus yielding more monodisperse particles while being more stable. They proved that protein concentration, as a marker for VLP stability, remained consistent for 12 months in the optimal buffer compared to NaNO_3_ without Tris, while in the latter the protein concentration decreased over time, indicating VLP degradation. Other than that, in the study by Yao et al. (2019) mentioned earlier, besides improved thermal stability, yields of aRNA using Qβ capsid were approximately 100 times higher than the reported yield using MS2 capsid in Pasloske and his team’s initial publication on aRNA.

## Closing Summary

Armored RNA (aRNA) technology has matured from a clever molecular trick into a platform with broad implications for public health. Initially developed to overcome the fragility of RNA controls in molecular assays, aRNA’s protective capsid now represents one of the most reliable tools for ensuring assay accuracy under the demanding conditions of pandemic diagnostics. The COVID-19 pandemic underscored how delays in establishing trustworthy diagnostic workflows can cripple early response efforts, of which aRNA’s speed of development and resilience could help close this gap in future outbreaks.

The core strengths of aRNA, exceptional nuclease resistance, safety through non-infectious design, and adaptability across RNA and DNA viral targets, have made it indispensable in molecular testing. Its proven use as internal process controls and external controls in RT-qPCR and RT-LAMP assays illustrate both its practicality and scalability. However, the technology is not static. Research into alternative systems such as Qβ-based VLPs, plant-based expression systems, and cell-free synthesis points to a healthy diversification of production strategies. These innovations not only address yield and stability challenges but also hint at a more distributed, decentralized production model that could democratize access to diagnostic reagents during health emergencies.

Looking forward, aRNA should be viewed not only as a diagnostic tool but as a platform for biomedicine. The ability to encapsidate therapeutic RNAs, self-amplifying vaccine candidates, or even custom-engineered payloads suggests a broader utility that aligns well with precision medicine and rapid pandemic response. While challenges remain such as payload size limits and cost of high-purity production, ongoing engineering of capsid architecture, pac site optimization, and downstream process improvements are steadily expanding its potential. In our view, the next generation of aRNA systems will not simply support diagnostics but could act as rapid-response vehicles for vaccines and therapeutics, representing a flexible biotechnological infrastructure for pandemic preparedness.

In conclusion, the trajectory of aRNA reflects a transition from proof-of-concept to strategic necessity. Its robustness and versatility make it uniquely suited to the realities of global health crises, where speed, stability, and safety cannot be compromised. Continued investment in this platform may well determine how quickly and effectively the world can mobilize against the next pandemic threat.

## Figures and Tables

**Table 1.** List of viral target positive controls that armored RNA has been developed for

Publication year	Viral target	Production system	RT-PCR limit of detection (LOD)	Reference
1998	HIV-1	*E. coli* DH5α	Not tested	Pasloske et al. (1998)
1999	Hepatitis C	*E. coli* DH5α	Not tested	WalkerPeach et al. (1999)
2005	Enterovirus	Not specified	Not specified	Donia et al. (2005)
2006	Classical swine fever virus (CSFV), foot-and-mouth disease virus (FMDV), vesicular stomatitis virus (VSV)	Cell-free production	10^2^ to 10^3^ copies for CSFV, and 10^3^ to 10^4^ copies for FMDV, 10 to 10^2^ target copies for VSV	Hietala and Crossley (2006)
2007	Rebulla	*E. coli*	Not specified	Zhao et al. (2007)
2007	Influenza A, influenza B, SARS	*E. coli* BL21 (DE3)	10^1^ copies/μl of AR-2	Yu et al. (2008)
2008	SARS-CoV	*E. coli* DH5α	25 copies/reaction	Stevenson et al. (2008)
2009	HIV-1	*E. coli* BL21 (DE3)	50 copies/ml	Zhan et al. (2009)
2010	Influenza A, influenza B, RSV, H1N1	*E. coli* DH5α	Not reported	Hymas et al. (2010)
2011	EV71, CA16, pan-EV	*E. coli* DH5α	Not reported	Song et al. (2011)
2013	Influenza A (H1N1, H5N1, H9N2)	*E. coli* DH5α	10^0^–10^1^ copies/ml	Chen et al. (2013)
2013	Influenza A (H7N9)	*E. coli* BL21 (DE3)	Not reported	Sun et al. (2013)
2015	Foot-and-mouth disease	*Nicotiana benthamiana*	Not reported	Madi et al. (2015)
2015	Measles	*E. coli* BL21 (DE3)	Not reported	Zhang et al. (2015b)
2015	HBV, HPV	*E. coli* BL21 (DE3)	Not tested	Zhang et al. (2015a)
2016	MERS-CoV	*E. coli* BL21 (DE3)	Not reported	Zhang et al. (2016a)
2020	SARS-CoV-2	*E. coli* BL21 (DE3)	10^3^ copies/ml	Goncharova et al. (2021)
2021	Zika	*E. coli* BL21 (DE3)	Not reported	Lin et al. (2017)
2022	SARS-CoV-2	*Nicotiana benthamiana*	Not reported	Peyret et al. (2022)
2024	nOPV2 poliovirus	*E. coli* BL21 (DE3)	10^3^ copies/ml	Dolgova et al. (2024)
2025	Measles	*E. coli* BL21 (DE3)	10^3^ copies/ml	Chayeb et al. (2025)

**Table 2.** Comparison between bacterial, plant-based, and cell-free production systems

Characteristic	Bacterial systems	Plant-based systems	Cell-free production
Production speed	Fast (hours–1 day)	Slower (days for transient expression)	Very fast (hours, no growth)
Yield	High, scalable in fermenters	Moderate; variable per biomass	Lower but adequate for small batches
Cost	Low	Moderate–high	High per unit; economical only at small scale
Assembly complexity	Well-established but may need optimization	Handles complex VLPs; more variable	Highly controlled but technically demanding
Product quality & Stability	Good if purified; risk of endotoxin	Batch variability; plant contaminants possible	High control; must guard against RNase
Biosafety	Non-infectious, low risk	Low pathogen risk after purification	Extremely low risk; no living host
Scalability	Easy to scale to bioreactors	More space and resources needed; feasible	Limited by reagent cost and supply
Regulatory/Contaminants	Well understood; bacterial nucleic acids must be removed	Less established; possible plant compounds	Few biological contaminants; reagent purity critical
Storage & Shipping	Stable when frozen/lyophilized	Similar to bacterial system after purification	Most flexible; can be lyophilized, potentially room-temperature stable
